# Engineering a synthetic anaerobic respiration for reduction of xylose to xylitol using NADH output of glucose catabolism by *Escherichia coli* AI21

**DOI:** 10.1186/s12918-016-0276-1

**Published:** 2016-04-16

**Authors:** Andrew Iverson, Erin Garza, Ryan Manow, Jinhua Wang, Yuanyuan Gao, Scott Grayburn, Shengde Zhou

**Affiliations:** Hubei Provincial Cooperative Innovation Center of Industrial Fermentation, Key Laboratory of Fermentation Engineering (Ministry of Education), College of Bioengineering, Hubei University of Technology, Wuhan, 430068 PR China; Department of Biological Sciences, Northern Illinois University, DeKalb, IL 60115 USA; Current address: William Rainey Harper College, Palatine, IL 60142 USA; School of Life Science, Fujian Normal University, Fuzhou, Fujian 350002 PR China

**Keywords:** *E. coli*, NADH output, Reducing power, *sdhCDAB-sucABCD* operon, Synthetic respiration

## Abstract

**Background:**

Anaerobic rather than aerobic fermentation is preferred for conversion of biomass derived sugars to high value redox-neutral and reduced commodities. This will likely result in a higher yield of substrate to product conversion and decrease production cost since substrate often accounts for a significant portion of the overall cost. To this goal, metabolic pathway engineering has been used to optimize substrate carbon flow to target products. This approach works well for the production of redox neutral products such as lactic acid from redox neutral sugars using the reducing power NADH (nicotinamide adenine dinucleotide, reduced) generated from glycolysis (2 NADH per glucose equivalent). Nevertheless, greater than two NADH per glucose catabolized is needed for the production of reduced products (such as xylitol) from redox neutral sugars by anaerobic fermentation.

**Results:**

The *Escherichia coli* strain AI05 (Δ*frdBC* Δ*ldhA* Δ*ackA* Δ(*focA*-*pflB*) Δ*adhE* Δ*ptsG* Δ*pdhR::pflBp*_6_-(*aceEF-lpd*)), previously engineered for reduction of xylose to xylitol using reducing power (NADH equivalent) of glucose catabolism, was further engineered by 1) deleting *xylAB* operon (encoding for xylose isomerase and xylulokinase) to prevent xylose from entering the pentose phosphate pathway; 2) anaerobically expressing the *sdhCDAB-sucABCD* operon (encoding for succinate dehydrogenase, α-ketoglutarate dehydrogenase and succinyl-CoA synthetase) to enable an anaerobically functional tricarboxcylic acid cycle with a theoretical 10 NAD(P)H equivalent per glucose catabolized. These reducing equivalents can be oxidized by synthetic respiration via xylose reduction, producing xylitol. The resulting strain, AI21 (pAI02), achieved a 96 % xylose to xylitol conversion, with a yield of 6 xylitol per glucose catabolized (molar yield of xylitol per glucose consumed (Y_RPG_) = 6). This represents a 33 % improvement in xylose to xylitol conversion, and a 63 % increase in xylitol yield per glucose catabolized over that achieved by AI05 (pAI02).

**Conclusions:**

Increasing reducing power (NADH equivalent) output per glucose catabolized was achieved by anaerobic expression of both the *pdh* operon (pyruvate dehydrogenase) and the *sdhCDAB-sucABCD* operon, resulting in a strain capable of generating 10 NADH equivalent per glucose under anaerobic condition. The new *E. coli* strain AI21 (pAI02) achieved an actual 96 % conversion of xylose to xylitol (via synthetic respiration), and 6 xylitol (from xylose) per glucose catabolized (Y_RPG_ = 6, the highest known value). This strategy can be used to engineer microbial strains for the production of other reduced products from redox neutral sugars using glucose as a source of reducing power.

## Background

Promising technologies are continuing to be developed for the conversion of cellulosic biomass into value-added commodities via microbial fermentation [4]. In order to be an economically viable process, however, anaerobic rather than aerobic microbial fermentation will most likely be used to produce redox neutral and reduced products. The anaerobic process will achieve a high substrate to product conversion yield since substrates often account for a significant portion of the production cost. To this end, metabolic pathway engineering has been used to optimize carbon flow from biomass-derived sugars to final products at high yields through manipulation of enzyme levels by over-expression, addition and/or deletion of target pathway genes [21]. Nevertheless, the insufficient supply of reducing power NADH (nicotinamide adenine dinucleotide, reduced) equivalent output of sugar catabolism remains a significant challenge for the production of reduced products from redox neutral biomass derived sugars under anaerobic conditions.

Most, if not all, microbial species can obtain a fixed number of NADH from any given carbon source under anaerobic conditions (e. g. two NADH can be formed from glycolysis by *E. coli* grown anaerobically). While limited, these NADH outputs provide the reducing power for the reduction of metabolic intermediates into fermentation products. In nature, with the limited NADH available from catabolism, some microorganisms have evolved multiple fermentation pathways to produce a mixture of redox neutral, oxidized, and reduced products to achieve a balanced redox in the absence of a suitable terminal electron acceptor. For example, under anaerobic conditions, *E. coli* carries out a mixed acid fermentation using the two NADH from glycolysis, producing oxidized (formic acid and succinic acid), redox neutral (acetic acid and lactic acid), and reduced (ethanol) products [8]. Genetic engineering has been successfully used to divert carbon flow and the reducing power (NADH) to produce redox neutral products, such as lactic acid, with a 100 % theoretical yield [25, 27]. Nevertheless, NADH availability from glucose catabolism often limits the yield of reduced products by anaerobic *E. coli* fermentation [13].

Increasing NADH availability, at least in theory, will accordingly increase the yield of reduced product via anaerobic fermentation. In prior studies, alteration of the NADH/NAD ratios by growing cells on carbon sources of various oxidative states [2, 15], or through supplementation of alternative electron acceptors [11], did indeed increase the proportion of reduced products from mixed acid fermentation of *E. coli*. In addition, Berríos-Rivera [5] increased the intracellular NADH availability two-fold through heterologous expression of a NAD^+^-dependent formate dehydrogenase (regenerating NADH) from *Candida boidinii* in *E. coli*, which resulted in a significant shift to reduced product (ethanol) accumulation and a dramatic increase in the ethanol-to-acetate ratio [6]. Furthermore, Cirino et al. [7] increased the NADH output of glucose catabolism by using an *E. coli* mutant with an anaerobic functional pyruvate dehydrogenase (PDH). Improved xylitol (a reduced product) yield was achieved from xylose reduction using the NADH output of glucose metabolism by this mutant.

Previously, we engineered *E. coli* SZ420, a strain with a doubled reducing power output through anaerobic expression of pyruvate dehydrogenase (*aceEF-lpd*), establishing a homoethanol pathway (glucose = > glycolysis= > 2 NADH + 2 pyruvate = > anaerobically synthesized pyruvate dehydrogenase = > 2 acetyl-CoA + 4 NADH = > alcohol dehydrogenase = > 2 ethanol) [28, 29]. Subsequently, SZ420 was engineered for reduction of xylose to xylitol (via synthetic respiration) using the reducing power of glucose catabolism by: 1) deleting the alcohol dehydrogenase (*adhE*) gene; 2) deleting the glucose-specific PTS permease complex (*ptsG*) to remove catabolic repression and allow simultaneous glucose and xylose uptake; and 3) expressing the aldose reductase gene (*xylI*) from *C. boidinii* [13]. The resulting strain, AI05 (pAI02), achieved a xylose to xylitol conversion ratio of 1:0.72, and a yield of 3.6 xylitol (from xylose) per glucose catabolized, with acetate as a minor by-product.

In this study, we report further engineering of *E. coli* AI05 with increased NADH output from glucose catabolism for effective reduction of xylose to xylitol by: 1) completely blocking xylose from entering the pentose phosphate pathway through deletion of genes encoding for xylose isomerase (*xylA*) and xylulokinase (*xylB*); 2) activation of an anaerobic TCA (tricarboxylic acid) cycle through anaerobic expression of the *sdhCDAB-sucABCD* operon, which encodes for succinate dehydrogenase (*sdhCDAB*), the α-ketoglutarate dehydrogenase complex (*sucAB*), and succinyl-CoA synthetase (*sucCD*). The resulting strain, AI21 (pAI02), achieved a xylose-to-xylitol conversion ratio of 1:1, a yield of 6 xylitol per glucose catabolized, and lacked acetate by-product accumulation.

## Methods

### Strains, plasmids, media, and growth conditions

Bacterial strains, plasmids and primers used in this study are listed in Table 1. For plasmid and strain construction, cultures were grown in Luria-Bertani (LB) broth (g/L: tryptone 10, yeast extract 5, NaCl 5) or on LB plates (agar 15 g/L). For enzymatic and NAD/NADH assays, cultures were grown in mineral salts medium broth (g/L: KH_2_PO_4_ 3.5, K_2_HPO_4_ 5.0, (NH_4_)_2_HPO_4_, MgSO_4_:7H_2_O 0.25, CaCl_2_:2H_2_O .015, thiamine 0.0005, and 1 mL of trace metal stock) [27]. Antibiotics were included in the media as needed at the following concentrations: kanamycin and ampicillin, 50 μg/mL; chloramphenicol, 40 μg/mL.

**Table 1:** *E. coli* strains, plasmids, and primers used in this study

Strains	Relevant characteristics	Sources
B	Wild type	ATCC11303
SZ420	*E. coli* B, Δ*frdBC* Δ*ldhA* Δ*ackA* Δ(*focA*-*pflB*) Δ*pdhR::pflBp*_(6)_-(*aceEF-lpd*)	Zhou et al. [28]
AI03	*E. coli* SZ420, Δ*adhE*	Iverson et al. [13]
AI05	*E. coli* SZ420, Δ*adhE* Δ*ptsG*	Iverson et al. [13]
AI09	*E. coli* SZ420, Δ*adhE* Δ*ptsG* Δ*xylB*	This study
AI12	*E. coli* SZ420, Δ*adhE* Δ*ptsG* Δ*xylB* Δ*sdhCp::*Fnr box- *pflBp*_(6)_-(*sdhCDBA-sucABCD*)	This study
AI21	*E. coli* SZ420, Δ*adhE* Δ*ptsG* Δ*xylB* Δ*xylA* Δ*sdhCp::*Fnr box- *pflBp*_(6)_-(*sdhCDBA-sucABCD*)	This study
Plasmids		
pKD4	*bla*, *FRT-km-FRT*	Datsenko and Wanner [10]
pKD46	*bla*, *γ β exo* (red recombinase), temperature-conditional replicon	Datsenko and Wanner [10]
pFT-A	*bla, flp*, temperature-conditional replicon	Posfai et al. [19]
pUC19	*bla* cloning vector	NE Biolab
pSD105	PCR amplified 0.35 kb *pflB* promoter region (BamHI- *pflBp*_6_-HindIII) was inserted into pSD101 at BamHI and HindIII sites	Zhou et al. [29]
pAGI02	PCR amplified 0.966 kb *xylI* region from *C. boidinii* was inserted into pSD105 at *HindIII* site	Iverson et al. [13]
Primers^a^		
Δ*xylB* N-primer	atgtatatcgggatagatcttggcacctcgggcgtaaaagttattgtgtaggctggagatgcttc	This study
Δ*xylB* C-primer	ttacgccattaatggcagaagttgctgatagaggcgacggaacgtcatatgatatcctccttag	This study
Δ*xylA* N-primer	ccgcggcattacctgattatggagttcaatatgcaagcctattttggtgtaggctggagatgcttc	This study
Δ*xylA* C-primer	gttatttgtcgaacagataatggtttaccagattttccagttgttccatatgaatatcctccttag	This study
Integration primer 1	**ccgacaaactatatgtaggttaattgtaatgattttgtgaacagcctatactgccgccag** gtgtaggctggagctgcttc (used as N-terminal primer for amplifying FRT*-kan-*FRT-Fnr box*- pflBp*_(6)_-*sdhC*’)	This study
Integration primer 2	**gaaccggatggtctgtaggtccagattaacaggtctttgttttttcacatttcttatcat** *gtaacacctaccttct*gttgctgtgatatagaagac (used as C-terminal primer for amplifying FRT*-kan-*FRT*- pflBp*_6_-*sdhC’*)	This study
*rrsA* primer 1	cggtggagcatgtggtttaa (used for qt-PCR)	Nishino et al. [18]
*rrsA* primer 2	gaaaacttccgtggatgtcaaga(used for qt-PCR)	Nishino et al. [18]
*sdhC* primer 1	cgccagccgcccagcacag (used for qt-PCR)	This study
*sdhC* primer 2	ggtatggaaggtctgttccgtcagattggtatttacagccc (used for qt-PCR)	This study
*sucA* primer 1	cagggcggttgcttcaccatctcca (used for qt-PCR)	This study
*sucA* primer 2	gcggcacgaactctttaccattccacacc (used for qt-PCR)	This study

^a^ The underlined sequence of Δ*xylB* N-primer, Δ*xylA* N-primer and intergration primer 1 is corresponding to primer 1 of pKD4; The underlined sequence of Δ*xylB* C-primer and Δ*xylA* C-primer is corresponding to the primer 2 of pKD46; the bold sequence of integration primer 1 is corresponding to the −219 to −174 bp upstream region of *sdhC*; the bold sequence of integration primer 2 is corresponding to the +1 to +45 of the *sdhC* coding sequence; The italicized sequence of integration primer 2 is corresponding to the 16 bp ribosomal binding site of *pflB*

### Genetic methods

Standard methods were used for plasmid construction, transformation, electroporation, and PCR [16, 20]. Chromosomal gene deletions were constructed using procedures developed by Posfai et al. [19] and Datsenko and Wanner [10]. Briefly, hybrid primer pairs were designed as follows: part of the primer is complementary to the deletion target gene and part complementary to the antibiotic cassette (FRT-*kan*-FRT) of pKD4 [10]. The amplified DNA, using these primer pairs and pKD4 as the template, was purified and electroporated into *E. coli* AI05 or its derivative (transformed with pKD46) using a micropulser (Bio-rad laboratories). As a result, the target gene was replaced by the FRT-*kan*-FRT cassette through homologous recombination (double crossover), resulting in kanamycin-resistant colonies. After streak-plate purification, the isolated colonies were verified by PCR analysis. The antibiotic marker cassette (FRT-*kan*-FRT) that was integrated onto the chromosome was then removed through FRT site-specific recombination via a flipase (FLP recombinase encoded by pFT-A, a temperature-conditional helper plasmid [19]).

### Transcriptional fusion of the *sdhCDAB-sucABCD* operon

**Fig. 1:**
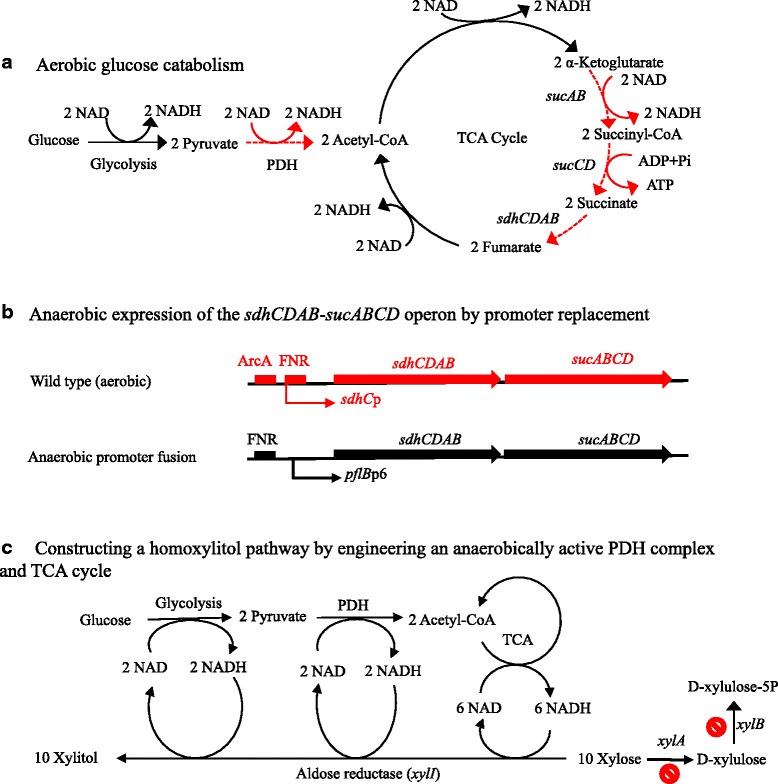
Engineering a homoxylitol pathway with an anaerobically active pyruvate dehydrogenase and TCA cycle. **a** NADH output of aerobic glucose catabolism; **b** Replacing the promoter of the *sdhCDAB-sucABCD* operon with an anaerobically functional promoter *pflB*p6; **c** Homoxylitol pathway with anaerobically active pyruvate dehydrogenase and TCA cycle but without active pentose phosphate pathway. Symbols: the dashed line in **a** indicates the step is not active under anaerobic condition; the prohibition sign in **c** indicates the step was blocked by deletion of the gene; ArcA, aerobic regulator binding box; FNR, anaerobic regulator binding box; *pflB*p6, a promoter of *pflB* (pyruvate formate-lyase). Genes and abbreviations: PDH, pyruvate dehydrogenase complex; TCA, tricarboxylic acid cycle; *sdhCDAB-sucABCD* operon, an eight gene operon that encodes for three enzymes: succinate dehydrogenase (*sdhCDAB*), the α-ketoglutarate dehydrogenase complex (*sucAB*), and succinyl-CoA synthetase (*sucCD*); *xylA*, xylose isomerase gene; *xylB*, xylulose kinase gene

The *pflB*p6-*sdhCDAB-sucABCD* transcriptional fusion (Fig. 1b) was constructed using previously described procedures [28, 29]. Hybrid primers were designed as follows (Table 1): the integration primer 1 consists of 45 bp corresponding to the −219 to −174 bp upstream region of *sdhC*, accompanied by a 20 bp sequence corresponding to primer 1 of pKD4; the integration primer 2 consists of 45 bp corresponding to +1 to +45 of the *sdhC* coding sequence, followed by the 16 bp ribosomal binding site of *pflB* and a 20 bp *pflB*p6 promoter sequence. A FRT-*kan*-FRT-*pflB*p6-*pflB*rbs product was amplified by PCR using the hybrid primer pair and pSD105 as the template, which contains the *pflB*p6 promoter and an upstream FNR-box (0.35 kb) derived from *E. coli* B [29]. Following purification, the amplified product (~2 kb) was electroporated into *E. coli* AI09 (transformed with pKD46). The resulting kanamycin-resistant recombinant colonies contained the transcriptional fusion of the FNR box, *pflB*p6 promoter, *pflB* ribosomal binding site, and the coding sequence of the *sdhCDAB-sucABCD* operon (Fig. 1b). After verification of this chromosomal gene fusion by analysis of the PCR products, the antibiotic marker (*kan*) was removed from the chromosome with the pFT-A encoded FLP recombinase as described previously.

### Enzymatic assays

Bacterial cells were grown (100 rpm or 200 rpm, 37 °C) to mid-log phase in 250 mL flasks containing 50 mL mineral salts broth supplemented with 50 mM succinate or α-ketoglutarate. These cells were pelleted, resuspended either in 10 mL 1× Tris buffer (α-ketoglutarate: 100 mM Tris, 2 mM dithiothreitol, pH 8.5) or potassium phosphate buffer (succinate dehydrogenase: 100 mM potassium phosphate, pH 7.4), cooled on ice for 20 min, and sonicated 3 times (10 s each round) using a Sonifier Cell Distributor W-350 (Branson Sonic Power Inc.). After centrifugation at 4 °C (5000 rpm), the sonicated cell broth supernatant was used as crude extract for the assays. The α-ketoglutarate dehydrogenase assay was performed using the following method [3]: 650 μL of Tris buffer (200 mM, 2 mM DTT, pH 8.5) and 150 μL of each of the following 10× components were added to a 1.5 mL quartz cuvette: α-ketoglutarate potassium salt (80.4 mM), 3-acetylpyridine adenine dinucleotide (20 mM), coenzyme A (0.87 mM), L-cysteine hydrochloride (20.6 mM). The reaction was initiated by adding 100 μL of the crude extract, with the absorbance read at 363 nm for 5 min using a UV-2401PC UV–VIS Recording Spectrophotometer (Shimadzu). All components without α-ketoglutarate potassium salt were used as the blank. One unit of enzyme activity was calculated as micromoles of 3-acetylpyridine adenine dinucleotide reduced per minute per mg of cell dry mass. The succinate dehydrogenase assay was performed using the following method [12]: 1.35 mL of potassium phosphate buffer (0.1 mM, pH 7.4) and 30 μL of each of the following 10× components were added to a 1.5 mL quartz cuvette: potassium cyanide (6.5 mg/mL buffer), 2,6-dichlorophenol indophenol (DCIP) (0.6 mg/mL), phenazine methosulfate (20 mg/mL), disodium succinate (54 mg/mL). The reaction was initiated by adding 30 μL of the crude extract, with the absorbance read at 600 nm for 8 min. All components without disodium succinate were used as the blank. One unit of enzyme activity was calculated as micromoles of DCIP reduced per minute per mg of cell dry mass. Assays were performed in triplicate.

### NADH/NAD assay

The NADH/NAD concentrations were analyzed using adapted methods of Wimpenny and Firth [26]. Bacterial cells were grown to ~1.0 OD_550_ in LB broth (50 mL) containing 2 % glucose at 37 °C under aerobic and oxygen limiting conditions. These cells were pelleted, and either treated with 300 μL HCl (200 mM, pH 1.5) for NAD extraction or with 300 μL KOH (200 mM, pH 11.5) for NADH extraction. The cells were then incubated at 50 °C for 10 min, cooled to 4 °C, and then neutralized by adding 300 μL either 100 mM NaOH (for NAD) or 100 mM HCl (for NADH). After centrifugation, the supernatants were used for the NADH/NAD assays which were performed in a 1.5 ml cuvette as follows: 400 μL mixed solution (maintained at 30 °C) containing equal amount of 3-(4,5-dimethyl-2-thiazolyl)-2,5-diphenyl-2H-tetrazolium bromide (4.2 mM), EDTA (40 mM), Tris (1 M, pH 8.0), and 95 % ethanol; 300 μL H_2_O; 200 μL phenazine ethosulfate (33.2 mM); and 50 μL sample supernatant. The reaction was initiated by adding 50 μL yeast alcohol dehydrogenase II (500 U/mL), and the absorbance was read at 570 nm for 5 min using a UV-2401PC UV–VIS Recording Spectrophotometer (Shimadzu). All components except ethanol were used as the blank. All assays were performed in triplicate.

### Quantitative real-time PCR

*E. coli* SZ420 and its derivatives were grown to ~1.5 OD_550_ in 250 ml screw-cap flasks containing 100 ml mineral salts media broth supplemented with either 50 mM glucose, 50 mM succinate, or 50 mM α-ketoglutarate (37 °C, shaking at 100 rpm). Bacterial cells (40 ml) were pelleted at 4 °C, resuspended by vortexing in 1 ml Tris-EDTA buffer (10 mM, pH 8.0, 0.1 mM EDTA, 1 mg of lysozyme), and mixed with 5 μl of 10 % SDS at 25 °C. From the cell suspension, 100 μL was extracted and total RNA was isolated using the PureLink RNA mini kit (Invitrogen) as described for bacterial cells. Extracted RNA was treated with RQ1 RNase-Free DNase (Promega Corp., Madison, WI) to remove residual chromosomal DNA. The RNA was then used for cDNA synthesis and qPCR analysis of *sdhCDAB-sucABCD* expression using the methods described previously [25, 29].

### Fermentations

For anaerobic cell growth and fermentations, the culture were inoculated in media that became essentially anaerobic as the growing cells consumed the small amount of oxygen present in the media, rather than by inoculating cells into anaerobic media under strictly anaerobic conditions. Specifically, seed cultures were prepared by inoculating a single colony from a fresh plate into 50 ml mineral salts broth containing 2 % glucose (kanamycin added for maintaining plasmid pAI02) and incubating (30 °C, 100 rpm) to ~2.0-4.0 OD_550_. After centrifugation, cell suspensions were used to inoculate (initial 0.05 OD_550_) 250 ml screw-cap flasks containing 100 ml mineral salts medium supplemented with 5 g L^−1^ glucose, 15 g L^−1^ xylose and 100 μl kanamycin. The flasks were then sealed by rubber caps. The fermentations were carried out in triplicate at 30 °C, 100 rpm shaking, and supplementation of 20 μl kanamycin every 24 h to maintain the plasmid. Samples were taken every 24 h for analysis of cell mass and concentration of sugars and fermentation products.

### Resting cell fermentation

Methods were adapted from previously described procedures [7]. Seed cultures were prepared by inoculating a single colony from a fresh plate into 100 ml mineral salts medium broth containing 2 % glucose, 1 % xylose, and 100 μl kanamycin and then incubated (30 °C, 100 rpm) until ~2.0-4.0 OD_550_. After centrifugation, cells were inoculated (initial 2.0 OD_550_) into 250 ml screw-cap flasks containing 50 ml modified mineral media (lacking ammonium phosphate, no new amino acids and enzymes will be synthesized) supplemented with 5 g L^−1^ glucose, 20 g L^−1^ xylose, and 50 μl chloramphenicol (inhibiting protein synthesis). Resting cell cultures were maintained at 30 °C and 100 rpm shaking for 48 h. Samples were taken every 24 h for analysis of cell mass and the concentration of sugars and fermentation products [13].

### Analyses

Cell mass was estimated by measuring the optical density at 550 nm (1.0 ml cells of 1.0 OD_550nm_ was approximately 0.33 mg dry weight) using a Unico1100 spectrophotometer with a round culture tube (1 cm diameter) as a cuvette [30]. The concentrations of sugars and fermentation products were determined by using high performance liquid chromatography (Waters HPLC) equipped with dual λ absorbance and refractive index detectors. Products were separated by using a Bio-Rad HPX 87H column with 4 mM H_2_SO_4_ as the mobile phase (10 μl injection volume, 0.4 ml/min, 45 °C) [25].

## Results and discussion

### Deletion of the *xylB* gene improved the xylose to xylitol conversion ratio

*E. coli* AI05 (pAI02) was previously engineered for the reduction of xylose to xylitol using the NADH output from glucose catabolism. The fermentation results of AI05 suggested that significant amounts of xylose were metabolized through the pentose phosphate pathway without being reduced to xylitol, representing a significant “substrate loss” [13]. Further analysis showed that the metabolized xylose was at least partially converted into acetate as a by-product [1, 13] (Fig. 1c). To prevent xylose loss and minimize acetate by-product accumulation, the *xylB* gene encoding for xylulokinase was deleted from AI05, resulting in strain AI09.

**Fig. 2:**
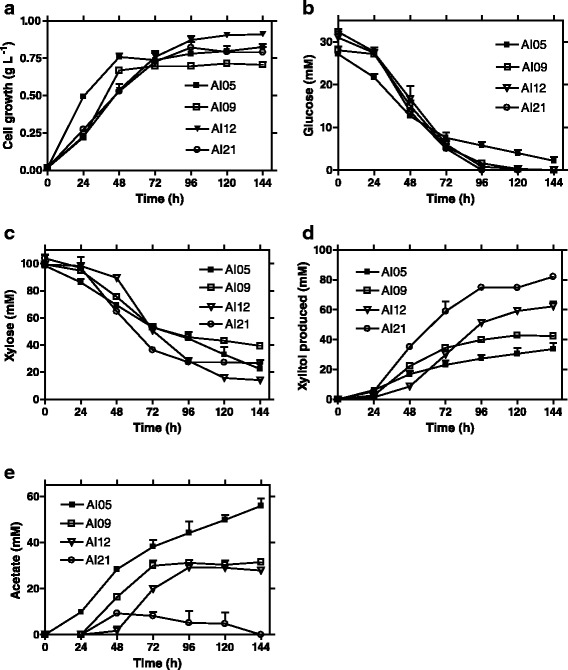
Xylitol fermentation from glucose (5 g L^−1^) and xylose (15 g L^−1^) mixture. **a** Cell growth; **b** Glucose utilization; **c** Xylose utilization; **d** Xylitol production; **e** Acetate by-product accumulation. Symbols for strain: filled square, AI05; open square, AI09; open triangle, AI12; open cycle, AI21

Xylose is still expected to be transported into AI09 cells via the XylE transporter, converted to D-xylulose by XylA, but blocked from further metabolism through the pentose phosphate pathway because *xylB* deletion blocks D-xylulose to D-xylulose-5 phosphate conversion (Fig. 1c). To evaluate the impact of the *xylB* deletion, fermentations were compared using AI05 and AI09 (Fig. 2). The results showed that the *xylB* deletion likely prevented some xylose loss from metabolism via the pentose phosphate pathway. During the 144 h fermentation, compared to that of AI05, AI09 produced more xylitol (42.5 vs 34 mM) and less acetate (31 vs 56 mM), but used an equivalent amount of xylose (60 vs 64 mM), resulting in an increased xylose to xylitol conversion ratio (1:0.71 vs 1:0.55). Nevertheless, the *xylB* deletion resulted in D-xylulose accumulation (data not shown); meaning some of the substrate was lost as an intermediate of the pentose phosphate pathway.

### Anaerobic expression of the *sdhCDAB-sucABCD* operon by promoter replacement

Although the *xylB* gene deletion improved the xylose to xylitol conversion ratio, the observed molar yield of xylitol per glucose metabolized (Y_RPG_ = 3.6) of AI09 in resting cell fermentation was expected to be similar to the one obtained by the parent AI05 (Y_RPG_ = 3.68) because a maximal 4 NADH output per glucose catabolized remained the same in both strains. Although this Y_RPG_ was comparable to the one reported in literature [1], the majority of the potential reducing power of glucose catabolized remained in the excess acetate due to the fact that the anaerobically growing *E. coli* cells have an incomplete TCA cycle for acetyl-CoA oxidation (partial oxidative and reductive branches). Furthermore, the reductive branch had been blocked by the *frdBC* deletion in our engineered strain AI05 [13, 29], making the oxidative branch irrelevant. The acetyl-CoA derived from pyruvate oxidation by the anaerobically transcribed pyruvate dehydrogenase complex was unable to be oxidized through TCA cycle, resulting in accumulation of acetate as a by-product. Our hypothesis is that a theoretical Y_RPG_ of 10 could be achieved if all of the reducing power had been extracted from glucose catabolism with the anaerobically active pyruvate dehydrogenase complex and a complete TCA cycle (10 NADH output per glucose catabolized for reduction of 10 xylose to 10 xylitol) (Fig. 1a and c).

Although the enzymatic activity has not been measured, the IcdA (isocitrate dehydrogenase) and AcnAB (isocitrate hydrolyase) should be active (at least in some degree) in our engineered strain because the parent strain can grow anaerobically in glucose minimal medium without supplement of glutamate (data not shown). The incomplete TCA cycle is probably due to the anaerobic repression of the *sdhCDAB-sucABCD* operon, which encodes for succinate dehydrogenase (*sdhCDAB*), the α-ketoglutarate dehydrogenase complex (*sucAB*), and succinyl-CoA synthetase (*sucCD*), three key enzymes needed for a complete TCA cycle [9, 23] (Fig. 1a, broken line). Although there is no guarantee, but very likely, a functional TCA cycle can be established, at least in some degree, by anaerobic expression of *sdhCDAB-sucABCD* operon. Therefore, a synthetic respiration pathway can be established for reduction of xylose to xylitol, using (theoretic 10) NADH from glucose catabolism via glycolysis, anaerobic expressed pyruvate dehydrogenase and functional TCA cycle.

To enable an anaerobically functional TCA cycle, chromosomal replacement of the native aerobic promoter of the *sdhCDAB-sucABCD* operon with the highly anaerobically functional promoter of the *pflB* gene (*pflB*p6) was performed. In the past, the *pflB*p6 promoter has been proven quite efficient in the expressing pyruvate dehydrogenase complex (*aceEF-lpd*, an aerobic operon) under anaerobic conditions [28, 29]. In addition, an upstream FNR (DNA-binding transcriptional regulator) box and the *pflB* ribosomal binding site were included in the promoter replacement for maximal expression of the *sdhCDAB-sucABCD* operon. The resulting strain was designated AI12 (Fnr box-*pflBp*_(6)_-*sdhCDAB-sucABCD*) (Fig. 1b).

The functionality of the transcriptional fusion of *pflB*p6 and the *sdhCDAB-sucABCD* operon engineered in *E. coli* AI12 was initially analyzed by quantitative PCR of the *sdhC* and *sucA* transcripts. The results showed that there was a 97, 15, and 10-fold increase in *sdhC* transcripts in AI12 cells grown on glucose, succinate, and α-ketoglutarate, respectively, compared to that of the control. qPCR analysis of the *sucA* transcripts revealed a similar trend of higher expression in AI12 than that of the control. These results confirmed that the *pflBp6* promoter allowed for effective expression of the *sdhCDAB-sucABCD* operon under oxygen-limiting conditions.

The transcriptional fusion of *pflB*p6 and *sdhCDAB-sucABCD* was further evaluated by analysis of the activities of α-ketoglutarate dehydrogenase and succinate dehydrogenase. The observed succinate dehydrogenase activity of AI12 displayed an 85 and 73 % increase for cells grown in glucose and succinate, respectively, compared to that of the control strain. Similarly, the observed α-ketoglutarate dehydrogenase activity displayed a 68 % increase in AI12 over that of the control strain.

### Anaerobically functional TCA cycle increased NADH output and xylitol yield per glucose catabolized

The enhanced enzymatic activities of α-ketoglutarate dehydrogenase and succinate dehydrogenase would allow acetyl-CoA oxidation via TCA cycle (Fig. 1a) and resulting in an improved NADH output from glucose catabolism. To evaluate the improvement in the NADH output of the engineered strain, AI12 was compared to its parent strain for cellular concentrations of NADH, NAD, and NADH/NAD ratios by growing cells in sealed screw-cap Erlenmeyer flasks containing 2 % glucose mineral salts medium. Subsequent assays showed a clear increase in the cellular NADH concentration of AI12 (0.0945 mM g DW^−1^) compared to that of the parent strain (0.0733 mM g DW^−1^). The calculated NADH/NAD ratios showed that there was about a 45 % increase in reducing power output in AI12 (0.7875) compared to that of the control (0.5414). These results are comparable to NADH/NAD ratios observed in *arcA* deletion mutants grown under similar conditions [22]. These results demonstrated that it is possible to increase the potential redox output of the TCA cycle (under anaerobic growth condition) without having to remove the ArcA (transcriptional regulator) and FNR global regulators. One issue still remains from the performed reducing power assays, the loss of reducing power from NADH oxidation by NADH dehydrogenases. However, there is still a clear indication that the anaerobic expression of the *sdhCDAB-sucABCD* operon increases the reducing power output.

Theoretically, the increased NADH output of glucose catabolism in AI12 would be used to reduce additional xylose to xylitol, resulting in an increase in xylitol yield per glucose catabolized. To test this hypothesis, xylitol fermentations were carried out by the engineered strains carrying plasmid pAI02 using mineral salts medium supplemented with glucose/xylose mixtures (5 g L^−1^ glucose, 15 g L^−1^ xylose) (Fig. 2). During the 144 h fermentation, strain AI12 used a similar amount of glucose (~30 vs 28 mM) (Fig. 2b), more xylose (~85 vs 60 mM) (Fig. 2c), and produced a significantly higher concentration of xylitol (63 vs 42 mM) (Fig. 2d) compared to that of the control strain, AI09. Based on these results, the calculated xylitol yield per glucose catabolized in AI12 (Y_RPG_ = 2.1) is significantly higher than that of AI09 (Y_RPG_ = 1.5), which suggests that at least some of the acetyl-CoA was oxidized through TCA cycle, generating extra NADH for xylose reduction. Nevertheless, the xylose to xylitol conversion ratio of AI12 (1:0.74) is similar to the one achieved by AI09 (1:0.72), suggesting that significant amounts of xylose was still lost as D-xylulose.

### Deletion of the *xylA* gene resulted in a 1:1 xylose to xylitol conversion

To improve the xylose to xylitol conversion ratio and minimize xylose loss as D-xylulose, the *xylA* gene, the first gene in the pentose phosphate pathway for xylose metabolism (Fig. 1c), was deleted from AI12, resulting in strain AI21. This deletion channeled all xylose into xylitol production as confirmed by AI21 fermentations (Fig. 2). Approximately 77 mM xylose was metabolized (Fig. 2c) to produce ~82 mM xylitol (Fig. 2d), suggesting an approximate 1:1 xylose to xylitol conversion in AI21. During this period, ~32 mM glucose was used as a source of reducing power. The calculated Y_RPG_ of AI21 was 2.56, although the actual Y_RPG_ would be over 3 if the glucose used for cell growth (0.79 g L^−1^) was considered (Table 2). It is interesting to note that a significantly decreased acetate accumulation by AI21 strain than that of AI12. This might be attributed to the re-use of acetate by conversion to acetyl-CoA (via acetate synthase (*acs*)) in AI21 (Fig. 2e) when glucose was completely metabolized after 96 h fermentation (Fig. 2b). In AI12 fermentation, however, acetate was not re-used because there was some glucose available until 144 h, resulting in accumulation of acetate.

**Table 2:** Summary of *E. coli* AI05 (pAGI02) and AI21 (pAGI02) fermentations ^a^

Parameters	AI05		AI21	
	Batch	Resting cell	Batch	Resting cell
Growth (g L^−1^)	0.825 ± 0.02	0	0.79 ± 0.024	0
Glucose used (mM)	24 ± 0.89	7.6 ± 0.05	32 ± 0.33	7.5 ± 0.63
Xylose used (mM)	75 ± 6.63	39 ± 0.2	77 ± 0.11	47 ± 0.28
Xylitol produced (mM)	34 ± 3.63	28 ± 0.68	82 ± 0.85	45 ± 0.35
Acetate produced (mM)	56 ± 3.04	18 ± 1.43	0	1 ± 0.63
Y_RPG_^b^	1.86	3.68	3.09	6.0
Xylitol produced/xylose used	0.45	0.72	1.06	0.96
Carbon recovery (%) ^c^	72	81	77	81

^a^ The data refers to that obtained at the end of fermentation (48 h for resting cell; 144 h for batch). A 0.5 % glucose and 1.5 % xylose sugar mixture was used for batch fermentation; while a 0.5 % glucose and 2 % xylose sugar mixture was used for resting cell fermentation

^b^ The Y_RPG_ was calculated from the total xylitol produced (mM) divided by the total glucose used (mM). For batch fermentation, the glucose used for cell growth was deducted from the total glucose consumed, yielding a Y_RPG_ of 1.86 and 3.09 for AI05 and AI21, respectively

^c^ The carbon recovery was calculated based on two assumptions: 1) the amount of CO_2_ produced (mM) equals the amount of acetate produced (mM); 2) the carbon weight accounts for 50 % of the cell mass

The Y_RPG_ of 2.56 achieved by AI21 was greater than that of other parent strains, illustrating that greater reducing power output does increase fermentative production of a reduced product. Though this Y_RPG_ value is approximately one-third of the theoretical potential of our engineered strain, this is the highest reported molar yield of xylitol produced per glucose consumed in batch cultures grown either aerobically or anaerobically [7, 17, 24].

### Resting cell fermentation

To evaluate the maximal NADH output potential of glucose catabolism by the engineered strain AI21, resting cell fermentations were performed using an equivalent cell density of OD_550_ 2.0 (0.67 g L^−1^ cell mass) in a screw-cap flask filled completely with modified mineral salts medium containing a glucose (5 g L^−1^) and xylose (20 g L^−1^) mixture, and chloramphenicol. This culture environment allowed cells to be metabolically active with the already available enzymes, but incapable of growth due to the lack of a nitrogen source and inhibition from chloramphenicol (no new enzyme synthesized). During resting cell fermentation, the observed Y_RPG_ values exceeded those of batch fermentations (Table 2). AI21 achieved an Y_RPG_ of 6, which is over 60 % higher than that achieved by the parent strain AI05 (Y_RPG_ of 3.68). In addition, there was no loss of xylose to by-products such as xylulose or acetate, confirming the 1:1 xylose to xylitol conversion. Furthermore, the apparent Y_RPG_ of 6 achieved by AI21 is significantly greater than the maximum previously reported by Cirino et al. (Y_RPG_ 4.7) (2006).

It is worthy to note that when cell growth is restricted, the theoretical maximum value of Y_RPG_ is 10, correlating with the maximum yield of 10 NADH from the complete oxidation of a molecule of glucose (Fig. 1c). Our most efficient strain, AI21, achieved 60 % of the theoretical maximum Y_RPG_. Since there was little acetate production (~1 mM) during resting cell fermentation, acetate accumulation was not attributed to the “missing NADH” per glucose catabolized (10 NADH equivalent potentially). Two possible outcomes of the “missing NADH” would be: 1) the oxidation of NADH through the electron transport system, although this pathway shouldn’t be active under anaerobic conditions; 2) the conversion of NADH to NADPH by the transhydrogenase or NADH kinase, although NADPH was reported to be the preferred reducing power used by the aldose reductase of *C. boidinii* (*xylI*) [14].

## Conclusion

The *E. coli* strain AI05 (pAI02) previously engineered for reduction of xylose to xylitol (via synthetic respiration) using the reducing power output from anaerobic glucose catabolism, was further improved by: 1) deleting the *xylAB* operon to block xylose loss through the pentose phosphate pathway, achieving a 100 % reduction of xylose to xylitol; 2) anaerobic expressing of the *sdhCDAB-sucABCD* operon to allow acetyl-CoA oxidation via TCA cycle, generating a theoretical 10 NADH output from the catabolism of one glucose for the reduction of 10 xylose to 10 xylitol. The resulting *E. coli* strain AI21 (pAI02) achieved an actual 100 % reduction of xylose to xylitol, and 60 % of the theoretical maximum xylitol yield per glucose catabolized (Y_RPG_ = 6). Nevertheless, an Y_RPG_ of 6 is the highest known value reported in literature. Further improvements in the xylitol yield could be achieved by enhancing the conversion of NADH to NADPH, the preferred reducing power of the aldose reductase of *C. boidinii* (*xylI*) [14]. In addition, this strategy can be used to engineer microbial strains for the homofermentative production of other reduced products from pentose sugars using glucose as a source of reducing power.

### Ethics

Not applicable.

### Consent to publish

Not applicable.

## Abbreviations

*aceEF-lpd*: pyruvate dehydrogenase operon
*ackA*: acetate kinase
AcnAB: isocitrate hydrolyase
*adhE*: alcohol dehydrogenase
ArcA: transcriptional dual regulator
DCW: dry cell weight
FLP: flipase
FNR: DNA binding transcriptional dual regulator
*frd*: fumarate reductase
FRT: flipase recognition target
HPLC: high pressure liquid chromatography
IcdA: isocitrate dehydrogenase
LB: Luria Bertani Broth
*ldhA*: D-lactate dehydrogenase
NAD: nicotinamide adenine dinucleotide
NADH: reduced form of nicotinamide adenine dinucleotide
OD_550_: optical density at 550 nm
PCR: polymerase chain reaction
PDH: pyruvate dehydrogenase complex
*pdhR*: repressor of pyruvate dehydrogenase operon
*pflB*: pyruvate formate lyase
*pflB*p6: promoter 6 of pyruvate formate lyase
pFT-A: plasmid containing the flipase gene
pKD46: plasmid containing the red recombinase system
PTS: phosphoenolpyruvate phosphotransferase system for sugar transport
*ptsG*: subunit of glucose PTS permease
*sdhCDAB-sucABCD* operon: encoding for succinate dehydrogenase, α-ketoglutarate dehydrogenase and succinyl-CoA synthetase
TCA: tricarboxylic acid
*xylA*: xylose isomerase
*xylB*: xylulokinase
*xylE*: xylose/proton symporter
*xylI*: aldose reductase
Y_RPG_: molar yield of xylitol per glucose consumed
